# A De-*O*-acylated Lipooligosaccharide-Based Adjuvant System Promotes Antibody and Th1-Type Immune Responses to H1N1 Pandemic Influenza Vaccine in Mice

**DOI:** 10.1155/2016/3713656

**Published:** 2016-11-07

**Authors:** Ji In Ryu, Shin Ae Park, Seo Ri Wui, Ara Ko, Ji Eun Han, Jung Ah Choi, Man Ki Song, Kwang Sung Kim, Yang Je Cho, Na Gyong Lee

**Affiliations:** ^1^Department of Bioscience and Biotechnology, Sejong University, Seoul, Republic of Korea; ^2^Laboratory Science Division, International Vaccine Institute, Seoul, Republic of Korea; ^3^R&D Center, EyeGene, Seoul, Republic of Korea

## Abstract

Vaccine adjuvants are agents that are used to promote immune responses to vaccine antigens and thereby to enhance the protective efficacy of the vaccines. In this study, we investigated the adjuvant activity of CIA06, an adjuvant system that is composed of a toll-like receptor 4 agonist de-*O*-acylated lipooligosaccharide (dLOS) and aluminum hydroxide, on the H1N1 pandemic influenza vaccine Greenflu-S® in mice. CIA06 significantly enhanced influenza-specific serum IgG, hemagglutination-inhibition, and virus-neutralizing antibody titers, which eliminated vaccine dose-dependency in the antibody response. Mice immunized with the CIA06-adjuvanted Greenflu-S showed Th1-type-predominant cytokine profiles, and both CD4^+^ and CD8^+^ T cell responses were induced. Immunization of mice with the CIA06-adjuvanted vaccine reduced the mortality and morbidity of mice upon lethal challenges with influenza virus, and no excessive inflammatory responses were observed in the lung tissues of the immunized mice after viral infection. These data suggest that the dLOS-based adjuvant system CIA06 can be used to promote the immune responses to influenza vaccine or to spare antigen dose without causing harmful inflammatory responses.

## 1. Introduction

The influenza virus could cause pandemic as well as seasonal epidemic infections; thus, widespread vaccination is the most effective infection control measure. However, because of antigenic shifts and drifts in the influenza virus, influenza vaccines are manufactured annually, which often causes vaccine supply shortages, especially during pandemics. To overcome potential vaccine shortages, vaccine adjuvants are used to promote immune responses to vaccine antigens [1] and serve as a feasible means to reduce the antigen dose required to achieve optimal protection [2].

Various adjuvants have been used with influenza vaccines to potentiate vaccine efficacy. MF59, an oil-in-water emulsion adjuvant, was applied to the 2009 influenza A (H1N1) hemagglutinin vaccine, and the results indicated that higher antibody titers were elicited from the MF59-adjuvanted vaccine containing 1/4 or 1/2 of the standard human dose compared with the nonadjuvanted vaccine, demonstrating an antigen reduction effect in humans [3]. In addition, MF59 was applied to inactivated vaccine to obtain optimal immunogenicity in young children and the elderly [4]. Similarly, AS03, another oil-in-water emulsion adjuvant containing tocopherol, was also successfully applied to the H1N1 influenza vaccine [5, 6]. Toll-like receptor 4 (TLR4) agonists, such as monophosphoryl lipid A (MPL) or glucopyranosyl lipid adjuvant (GLA), when combined with alum or in oil-in-water formulation, also exhibited potential for use as adjuvant for influenza vaccines [7, 8].

De-*O*-acylated lipooligosaccharide (dLOS) is a TLR4 agonist that is derived from an* Escherichia coli* lipopolysaccharide (LPS) containing a short carbohydrate moiety [8, 9]. Unlike MPL or GLA, dLOS contains a core oligosaccharide with N-linked acyl groups on lipid A moiety [9]. Similar to MPL, dLOS induces the secretion of cytokines from murine peritoneal macrophages but demonstrates more potent activation of human monocytes and dendritic cells (DCs) [9]. dLOS, when combined with bacterial DNA fragments (designated as CIA07), significantly enhanced both humoral and cellular immune responses to hepatitis B virus surface antigen and influenza subunit vaccine [10, 11]. CIA07 also exhibited potent immunostimulating activity, which suggests its potential as a cancer immunotherapy [8]. A combination of dLOS and aluminum hydroxide (designated as CIA06) was also evaluated for adjuvant effects against human papillomavirus (HPV) L1 virus-like particles (VLPs) and anthrax protective antigen (PA) [12–15]. The results indicated that CIA06 effectively increased antibody titers to both vaccines and that the induced antibodies were effective in neutralizing HPV pseudovirus and anthrax lethal toxin, respectively [13–15]. Further, the enhancement of IgG and toxin-neutralizing antibody titers allowed the dose of PA antigen required for immunization to be reduced, which suggested a potential antigen sparing effect [14]. The CIA06 adjuvant was most effective in inducing immune responses to HPV L1 VLPs at a ratio of 1 : 50 between dLOS and alum, as evidenced by serum antibody titers, splenic IFN-*γ* secretion, and antigen-specific memory B cell responses [13]. These results indicated that CIA06 is capable of inducing both Th1- and Th2-type immune responses that persist for an extended period. Subsequently, the safety and immunogenicity of the CIA06-adjuvanted HPV vaccine were confirmed in a human trial (unpublished data).

In this study, we investigated the adjuvant activity of CIA06 on a commercial H1N1 pandemic influenza vaccine in mice. The results showed that CIA06 significantly increased the immunogenicity of the vaccine and enhanced the protection of mice against a lethal influenza virus challenge more than 20-fold, which demonstrated that the addition of CIA06 as an adjuvant could promote the protective efficacy of influenza vaccine and allow us to reduce influenza vaccine doses during an influenza pandemic.

## 2. Materials and Methods

### 2.1. Materials

Influenza virus A/California/07/2009 (H1N1) and mouse-adapted A/California/04/2009 (H1N1) virus strains were obtained from the Korea Centers for Disease Control and Prevention (Seoul, Korea) and the International Vaccine Institute (Seoul, Korea), respectively. The viruses were cultured in the allantoic cavity of embryonated eggs, harvested, and stored at −80°C until analysis. Madin-Darby canine kidney (MDCK) cells were acquired from American Type Culture Collection (ATCC, Manassas, VA, USA). Cell culture media and antibiotics were purchased from Welgene (Daegu, Korea), and fetal bovine serum (FBS) was purchased from Gibco/Invitrogen (Grand Island, NY, USA). Aluminum hydroxide (alum, Alhydrogel®) was purchased from Superfos Biosector (Frederikssund, Denmark), whereas squalene-based oil-in-water adjuvant AddaVax™ was obtained from InvivoGen (San Diego, CA, USA). Goat anti-mouse IgG antibody was obtained from Jackson Immuno Research Labs (West Grove, PA, USA) and Serotec (Kidlington, Oxford, UK), whereas purified anti-mouse CD4 and CD8 antibodies were purchased from BioLegend (San Diego, CA, USA). Mouse anti-influenza A nucleoprotein (NP) monoclonal antibody (mAb) and FITC-conjugated rat anti-myeloperoxidase (MPO) antibody were obtained from Millipore (Billerica, MA, USA) and Abcam (Cambridge, UK), respectively. Recombinant mouse IL-2 was acquired from R&D Systems (Minneapolis, MN, USA), and IFN-*γ* and IL-5 cytokine ELISA kits were obtained from BD Biosciences (San Diego, CA, USA) and R&D Systems.

### 2.2. Influenza Vaccine and Adjuvants

Greenflu-S (Green Cross, Yong In, Korea), the pandemic influenza A/California/07/2009 (H1N1) virus split vaccine, was used in this study. dLOS was prepared from an* E. coli* LPS mutant strain as previously described [9], quantified using the 2-keto-3-deoxyoctonate assay as previously described [16], and visualized on a silver-stained SDS-polyacrylamide gel. The adjuvant system CIA06 was prepared by mixing dLOS and alum in a 1 : 50 ratio [13].

### 2.3. Immunization of Mice

Specific pathogen-free 6-week-old female BALB/c mice were purchased from SLC (Hamamatsu, Japan) and randomly assigned into experimental groups consisting of three to six mice. The animals were immunized twice at a 2- or 3-week interval via intramuscular injection with Greenflu-S alone or in combination with alum (25 *μ*g) or CIA06 (0.5 *μ*g dLOS plus 25 *μ*g alum) in phosphate-buffered saline (PBS, pH 7.3). Control mice were given PBS or CIA06. Blood, lung, and spleen samples were collected from the animals two weeks after the second immunization. All animal experiments were reviewed and approved by the Animal Care and Welfare Committees of Sejong University and the International Vaccine Institute.

### 2.4. Enzyme-Linked Immunosorbent Assay (ELISA)

Influenza virus-specific antibody titers were measured on individual mouse sera by endpoint dilution ELISA as previously described by Han et al., with slight modifications [13]. Briefly, a 96-well immunoplate (Nunc, Roskilde, Denmark) was coated with influenza A/California/07/2009 (H1N1) virus at a concentration of 16 HA units/well and incubated overnight at 4°C. Following incubation, the wells were washed with PBS Tween (PBST), blocked with 1% bovine serum albumin (BSA) in PBS, and incubated with mouse serum that was 2-fold serially diluted with 1% BSA in PBS. Bound antibody was detected with horseradish peroxidase-conjugated goat anti-mouse IgG, IgG1, or IgG2a antibody for 1 h at 37°C followed by incubation with 3,3′,5,5′-tetramethylbenzidine substrate (BD Biosciences). The reaction was stopped and absorbance at 450 nm was measured using an Infinite M200 microplate reader (Tecan, Männedorf, Switzerland). The endpoint titer was defined as the highest serum dilution with an absorbance value two times greater than the nonimmune serum absorbance value, with the cut-off value set at 0.1. The geometric mean titers (GMT) were calculated from individual log_10_-transformed titers and were expressed as GMT ± standard deviation (SD) for each experimental group of six mice.

### 2.5. Determination of Serum Hemagglutination-Inhibition Antibody Titer

The hemagglutination-inhibition (HI) antibody titers of individual mouse sera were determined as previously described [17]. Serum samples were treated with a receptor-destroying enzyme (RDE, Denka Seiken, Tokyo, Japan) and heat-inactivated at 56°C for 30 min. The pretreated serum (50 *µ*L) was then transferred to a 96-well V bottom plate (Corning, NY, USA), twofold serially diluted with PBS, and mixed with 8 hemagglutinin units of viral suspension. Following incubation at room temperature for 1 h, a 50 *μ*L aliquot of 1% turkey red blood cell (RBC) suspension was added to each well, and the samples were incubated for an additional 30 min before the determination of hemagglutination. The HI antibody titer was defined as the reciprocal of the highest serum dilution that completely inhibited hemagglutination, and the results were expressed as the geometric means of values obtained from groups of six mice.

### 2.6. Virus-Neutralization Assay

In order to determine the virus-neutralizing antibody titers of mouse immune sera, individual serum samples (60 *μ*L) were treated with RDE, heat-inactivated, twofold serially diluted with PBS in a 96-well plate, and mixed with an equal volume of 100 TCID_50_ of the influenza virus. The mixtures were added to MDCK cells seeded in a 96-well plate at a density of 2.5 × 10^5^ cells/mL and cultured in a humidified CO_2_ incubator for three days. Cells were stained with crystal violet to determine the cytopathic effects or with anti-influenza A NP mAb to detect influenza virus NP protein as previously described [17].

### 2.7. Measurement of Cytokine Levels

Single cell suspensions were prepared from the spleens of immunized mice. Red blood cells were lysed, and the resulting splenocytes were resuspended in RPMI1640 supplemented with 10% FBS, 100 U/mL of penicillin, 100 *μ*g/mL of streptomycin, 0.5 ng/mL of recombinant mouse IL-2, 10 mM HEPES, and 50 *μ*M *β*-mercaptoethanol. Cells were seeded at a density of 2 × 10^6^ cells/mL in a 96-well culture plate and incubated in the absence or presence of Greenflu-S (2 *μ*g/mL) at 37°C for 72 h. The culture media were collected, and IFN-*γ* and IL-5 cytokine levels were measured by sandwich ELISA. Results are expressed as the means ± SD of values obtained from triplicate cultures that used two spleens each.

To determine the contribution of CD4^+^ and CD8^+^ T cells to IFN-*γ* production, T cell coreceptors were blocked by incubating cells with 1 *μ*g of purified anti-mouse CD4 or CD8 antibodies for 1 h before antigen stimulation. The levels of cytokines secreted from mouse splenocytes were measured.

### 2.8. Mouse Protection Assay

To evaluate the protective efficacy of nonadjuvanted and adjuvanted Greenflu-S, groups of five mice were immunized twice at a 2-week interval via intramuscular injection with Greenflu-S alone or in combination with adjuvants. Four weeks after immunization, mice were challenged intranasally with 10 LD_50_ of mouse-adapted influenza A/California/04/2009 virus and monitored for body weight change and survival for 15 days. The survival rates were determined by death or >25% loss in body weight, at which point the mice were humanely euthanized.

To determine the long term immunity induced by the vaccines, groups of six mice were immunized twice as above. A group of mice receiving AddaVax-adjuvanted Greenflu-S was included for comparison. Blood samples were collected by retroorbital puncture 4 and 24 weeks after the second immunization. Then, mice were challenged with 10 LD_50_ of influenza virus 26 weeks after immunization. Influenza virus specific IgG and HI antibody titers of individual sera were determined.

### 2.9. Measurement of Virus Titers in the Mouse Lung Tissues

Mouse lung tissue samples were collected 4 days after the viral challenge, homogenized in PBS containing antibiotics, and centrifuged at 12,000 ×g. Supernatants were 10-fold serially diluted, inoculated into 95% semiconfluent MDCK cells, and incubated at 37°C for 1 h. The culture media were then discarded, a low-temperature melting agarose solution was added over the top of the cells, and the cells were incubated at 37°C for 3 days. The cells were then fixed with 4% paraformaldehyde and stained with crystal violet, and the viral plaques that formed were counted.

### 2.10. Histology of Mouse Lungs

Lung tissues were fixed with 4% paraformaldehyde at 4°C for 24 h, embedded in paraffin, sectioned on a microtome (Leica Microsystems, Wetzlar, Germany), and stained with hematoxylin and eosin (H&E). To detect MPO expression and influenza virus, lung tissues were fixed with 4% paraformaldehyde, dehydrated in a gradient sucrose solution, and embedded in optimum cutting temperature (OCT) compound (Leica Microsystems). Cryosections approximately 5 *μ*m thick were generated using a Cryostat (CM-1850, Leica Microsystems), fixed in 80% cold acetone, and blocked for 1 h in PBS containing 5% rat serum, 3% BSA, 0.1% fish gelatin, 5 *μ*g/mL of anti-mouse CD16/CD32 antibody (BD Biosciences), and 0.1% Triton X-100. The tissues were stained with FITC-conjugated rat anti-MPO antibody or with mouse anti-influenza A NP mAb and eFluor®570-conjugated rat anti-mouse IgG2a antibody (eBioscience, San Diego, CA, USA). The tissues were counterstained with 4′,6-diamidino-2-phenylindole (DAPI, Lonza, Basel, Switzerland) and observed under a fluorescence microscope (DM2500, Leica Microsystems).

### 2.11. Measurement of Cytokine mRNA Levels of Mouse Lung Tissues

Mouse lung tissues were soaked in Easy-Blue reagent (Invitrogen, CA, USA), flash-frozen in liquid nitrogen, and stored at −70°C until analysis. Frozen tissues were thawed on ice and homogenized using a disperser (IKA, Staufen, Germany). Total cellular RNA was isolated from the lung homogenates using a Hybrid-R RNA purification kit according to the manufacturer's protocol (GeneAll, Seoul, Korea).

The mRNA levels of cytokine and chemokine genes were determined by semiquantitative reverse transcriptase-polymerase chain reaction (RT-PCR). The *β*-actin gene was included as an internal control. Template RNA (3 *μ*g) was reverse transcribed to cDNA using random hexamers and Moloney Murine Leukemia Virus Reverse Transcriptase (M-MLV RT, Invitrogen, Carlsbad, CA, USA), and the RT-reaction products were PCR amplified using gene specific primers (sequences are shown in Table S1, in Supplementary Material available online at http://dx.doi.org/10.1155/2016/3713656). Each PCR cycle comprised denaturation at 95°C for 30 s, annealing at 56°C for 45 s, and elongation at 72°C for 45 s. The RT-reactions and semiquantitative RT-PCR were performed for various cycles using a PTC-100 Thermo Cycler (Bio-Rad Laboratories, Philadelphia, PA, USA). The PCR products were resolved on agarose gels, and band images were captured using a Gel Logic 100 Imaging System (Eastman Kodak Company, Rochester, NY, USA) and analyzed by Molecular Imaging Software (Eastman Kodak Company). To obtain the intensity of the nonsaturated PCR band image, the images of 30 cycles were taken for TNF-*α*, IFN-*γ*, IL-6, IL-12, and IFN-*β*, and those of 25 cycles were taken for MCP-1 and RANTES. The mRNA levels of each gene were expressed as relative levels to those of the infected PBS control mice.

### 2.12. Statistical Analysis

SPSS 18.0 software (IBM, New York, USA) was used to perform statistical analysis. Differences among experimental groups were analyzed using one-way ANOVA with Tukey's multiple comparison test. Survival data for different mouse groups were analyzed using Kaplan-Meier survival curves and log-rank test. Repeated measures ANOVA followed by Bonferroni's posttest was used to analyze body weight changes for different mouse groups over time. *P* values of less than 0.05 were considered statistically significant.

## 3. Results

### 3.1. CIA06 Increased Serum Antibody Titers to Influenza Vaccine Greenflu-S

**Figure 1:**
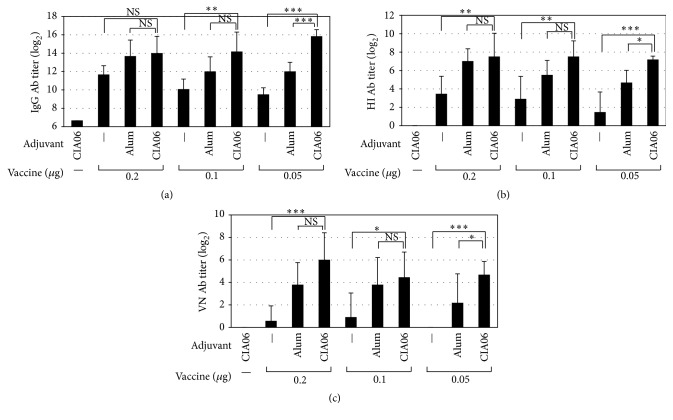
Increased serum antibody responses by CIA06-adjuvanted Greenflu-S. Mice (*n* = 6) were immunized twice at a 3-week interval with Greenflu-S alone or in combination with alum or CIA06. Control mice were given CIA06. Two weeks after the second immunization, blood samples were collected, and serum titers of influenza virus-specific IgG (a), HI antibody titers (b), and virus-neutralizing antibody titers (c) were determined. Statistical differences were analyzed by one-way ANOVA followed by Tukey's multiple comparison test. Results are presented as the GMT ± SD of titers obtained from six mice in each group. Data shown are representative of three independent experiments. ^*∗*^ *P* < 0.05; ^*∗∗*^ *P* < 0.01; and ^*∗∗∗*^ *P* < 0.001. NS, not significant.

The adjuvant effect of CIA06 on the influenza vaccine was evaluated using the pandemic influenza A/California/07/2009 (H1N1) virus split vaccine Greenflu-S. Mice were immunized with 0.2, 0.1, or 0.05 *μ*g vaccine doses, either alone or in combination with alum or CIA06, and influenza virus-specific serum total IgG antibody titers were assessed. In the groups of mice immunized with the nonadjuvanted vaccine, serum IgG antibody titers increased in a vaccine dose-dependent manner (Figure 1(a)). The addition of either alum or CIA06 to the vaccine enhanced the antibody titers compared to those of each corresponding nonadjuvanted vaccine dose group. CIA06 exhibited significantly higher adjuvant activity compared to alum in the animals given the 0.05 *μ*g vaccine dose (*P* < 0.001), which eliminated the vaccine dose-dependency. The adjuvant activity of dLOS alone was similar to that of alum (data not shown).

Next, the HI antibody titers of the mouse immune sera were measured. The results revealed that immune sera HI antibody titers correlated with the influenza virus-specific IgG titers (Figure 1(b)). The mice given the CIA06-adjuvanted vaccine were significantly higher in HI antibody titers compared to those given the nonadjuvanted vaccine regardless of vaccine dose (*P* < 0.01) and those given the alum-adjuvanted vaccine at 0.05 *μ*g vaccine dose (*P* < 0.05). Therefore, dose-dependency was not observed among the CIA06-adjuvanted vaccine groups. The ability of the immune sera to neutralize influenza virus was also assessed. In the sera of the mice immunized with the nonadjuvanted vaccine, virus-neutralizing activity was barely detectable (Figure 1(c)). All of the adjuvanted vaccine groups exhibited higher neutralizing activity compared to the corresponding nonadjuvanted vaccine groups, but the highest activity levels were apparent in the CIA06-adjuvanted vaccine group. Together, these results demonstrate that CIA06 was most effective in enhancing antibody responses to the influenza vaccine.

### 3.2. CIA06-Adjuvanted Greenflu-S Induces Th1-Type-Predominant Immune Responses

**Figure 2:**
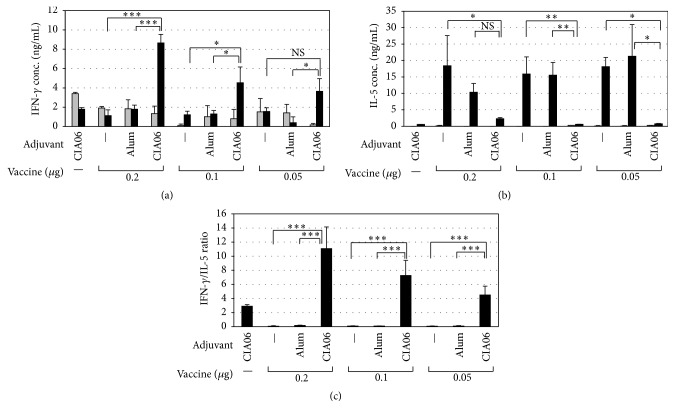
Th1-type-predominant immune response to CIA06-adjuvanted influenza vaccine. Splenocytes were isolated from the mice that had been immunized as described in Figure 1 and cultured for 72 h in the absence (■) or presence of Greenflu-S (■). The levels of IFN-*γ* (a) and IL-5 (b) in the culture media were determined by sandwich ELISA, and IFN-*γ* : IL-5 ratios were calculated for each group (c). Statistical differences were analyzed by one-way ANOVA followed by Tukey's multiple comparison test. Results are expressed as the means ± SD of values obtained from triplicate cultures that used two spleens each. Data shown are representative of three independent experiments. ^*∗*^ *P* < 0.05; ^*∗∗*^ *P* < 0.01; and ^*∗∗∗*^ *P* < 0.001. NS, not significant.

To determine the ability of adjuvants to promote Th1-type immune responses to influenza vaccine, splenic cytokine levels were measured. As shown in Figure 2(a), levels of vaccine-specific IFN-*γ* in the nonadjuvanted or alum-adjuvanted vaccine groups were the same as the level in the control group regardless of vaccine dose. Conversely, the IFN-*γ* levels in the CIA06-adjuvanted vaccine groups were greatly increased upon stimulation with Greenflu-S and appeared to be dependent on the vaccine dose. The splenic levels of IL-5 exhibited an inverse relationship to IFN-*γ* levels among the experimental groups (Figure 2(b)), and the ratios of IFN-*γ* to IL-5 were highest in the CIA06-adjuvanted vaccine group (Figure 2(c)). These data suggest that the CIA06-adjuvanted vaccine induces Th1-type-biased immune responses.

**Figure 3:**
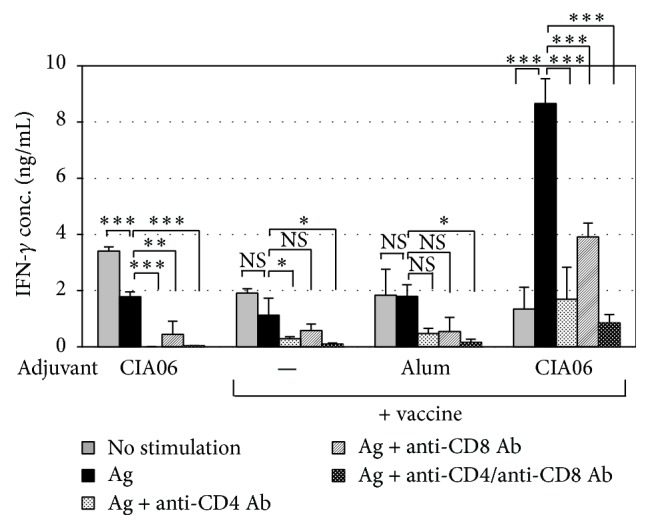
CIA06-adjuvanted influenza vaccine stimulates both CD4^+^ and CD8^+^ T cell responses. Splenocytes were isolated from the mice (*n* = 6) that had been immunized twice at a 3-week interval with nonadjuvanted or adjuvanted Greenflu-S (0.2 *μ*g) and stimulated with the vaccine for 72 h in the absence or presence of anti-CD4 and/or anti-CD8 antibodies. IFN-*γ* levels in the culture media were determined by sandwich ELISA. Statistical differences were analyzed by one-way ANOVA followed by Tukey's multiple comparison test. Results are expressed as the means ± SD of values obtained from triplicate cultures that used two spleens each. Data shown are representative of three independent experiments. ^*∗*^ *P* < 0.05; ^*∗∗*^ *P* < 0.01; and ^*∗∗∗*^ *P* < 0.001. NS, not significant.

T cell subsets responsible for vaccine-specific IFN-*γ* secretion were assessed by determining IFN-*γ* levels following the incubation of splenocytes with anti-CD4 antibody and/or anti-CD8 antibody (Figure 3). In the mice immunized with the CIA06-adjuvanted vaccine, anti-CD4 antibody treatment reduced IFN-*γ* secretion by 78% compared with untreated splenocytes, whereas anti-CD8 antibody resulted in a 55% reduction in IFN-*γ* secretion. IFN-*γ* secretion in cells treated with both antibodies was almost negligible. These results indicate that the CIA06-adjuvanted vaccine induced both CD4^+^ and CD8^+^ T cell responses.

### 3.3. CIA06 Promotes the Protective Efficacy of Greenflu-S against Lethal Infection with Influenza Virus in Mice

**Figure 4:**
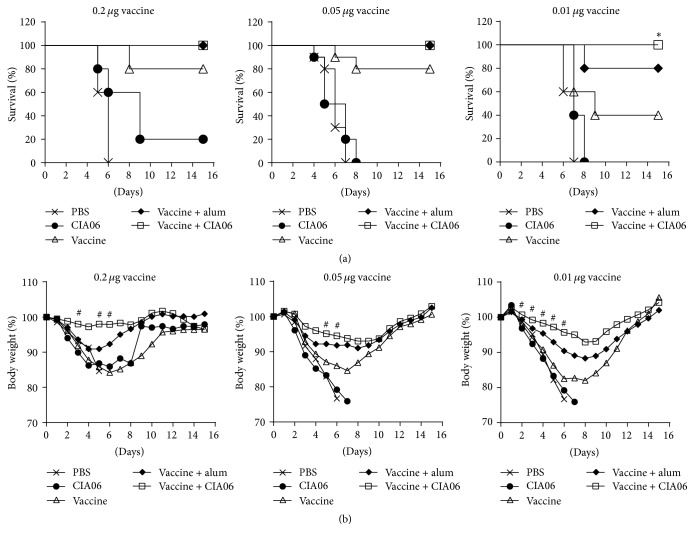
CIA06-adjuvanted Greenflu-S reduces the mortality and morbidity of mice upon lethal infection with influenza virus. Mice were immunized twice at a 2-week interval with 0.2, 0.05, or 0.01 *μ*g of nonadjuvanted or adjuvanted Greenflu-S. Control mice were given PBS or CIA06. Four weeks after immunization, mice were challenged with homologous influenza virus and monitored for survival (a) and change in body weight (b) for 15 days. Data shown in (b) are presented as the means that were obtained from each group of mice. Survival data for different mouse groups were analyzed using Kaplan-Meier survival curves and log-rank test. Repeated measures ANOVA followed by Bonferroni's posttest was used to analyze body weight changes for different mouse groups over time. Symbols *∗* and # indicate *P* < 0.05 and *P* < 0.008, respectively, when compared with the group given the nonadjuvanted vaccine.

The protective activity of the adjuvanted influenza vaccines was likewise evaluated. Mice immunized with 0.2 *μ*g of the adjuvanted or nonadjuvanted vaccine were challenged with a homologous influenza virus. The control mice receiving PBS or CIA06 alone exhibited 0% and 20% survival rates after influenza infection, respectively, whereas 80% of the mice immunized with the nonadjuvanted vaccine were protected against the viral challenge (Figure 4(a)). In contrast, all of the mice given the adjuvanted vaccines survived irrespective of the adjuvant type, which indicated 100% protective efficacy. However, differences in body weight change among the groups were detected. The mice given the CIA06-adjuvanted vaccine exhibited the lowest body weight loss percent (<3%, *P* < 0.008 versus the nonadjuvanted vaccine group), while mice receiving the alum-adjuvanted vaccine experienced body weight loss percentages as high as 10% (Figure 4(b)). Reducing the vaccine dose decreased protective efficacy; thus, the adjuvant effects were more striking. At doses of 0.01 *μ*g, the protective efficacy of the nonadjuvanted vaccine was only 40%, but the CIA06-adjuvanted vaccines provided complete protection against the viral challenge. The protective efficacy of the adjuvanted and nonadjuvanted vaccines at 0.05 *μ*g dose was between the protective efficacy of the vaccines at 0.2 *μ*g and that at 0.01 *μ*g doses.

**Figure 5:**
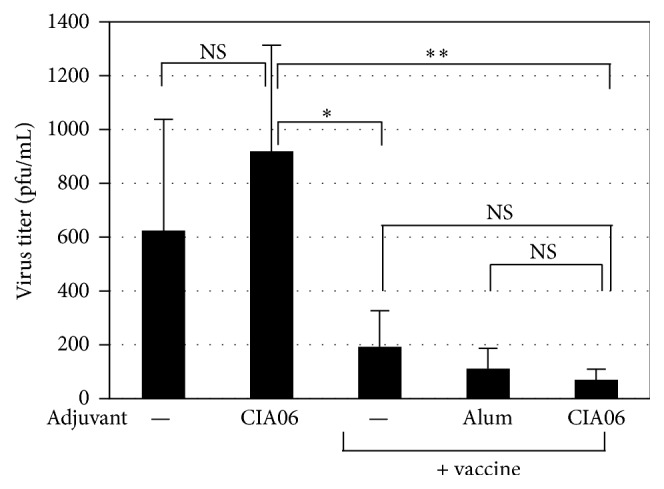
Decrease in lung virus titers of immunized mice after viral challenge. Mice (*n* = 4) were immunized twice with nonadjuvanted or adjuvanted Greenflu-S (0.2 *μ*g) and challenged with influenza virus. Control mice were given PBS or CIA06. Four days after infection, lung samples were collected, and virus titers were determined by plaque assays. Statistical differences were analyzed by one-way ANOVA followed by Tukey's multiple comparison test. Data are presented as the means ± SD of values that were obtained from each group of four mice. ^*∗*^ *P* < 0.05 and ^*∗∗*^ *P* < 0.01. NS, not significant.

The ability of the influenza vaccines to clear the virus in the lungs was evaluated in the mice given the 0.2 *μ*g influenza vaccine dose. Following two immunizations, the mice were challenged with a lethal dose of influenza virus, and virus titers in the lungs were measured 4 days after challenge. Virus titers in the lungs from mice immunized with the adjuvanted and nonadjuvanted vaccines were significantly lower compared with the lung virus titers of mice in the PBS and CIA06 adjuvant control groups. Further, among the vaccinated groups, the CIA06-adjuvanted vaccine was the most effective in clearing the virus from the lungs (Figure 5).

### 3.4. Persistence of the Protective Immunity Elicited by the CIA06-Adjuvanted Influenza Vaccine

**Figure 6:**
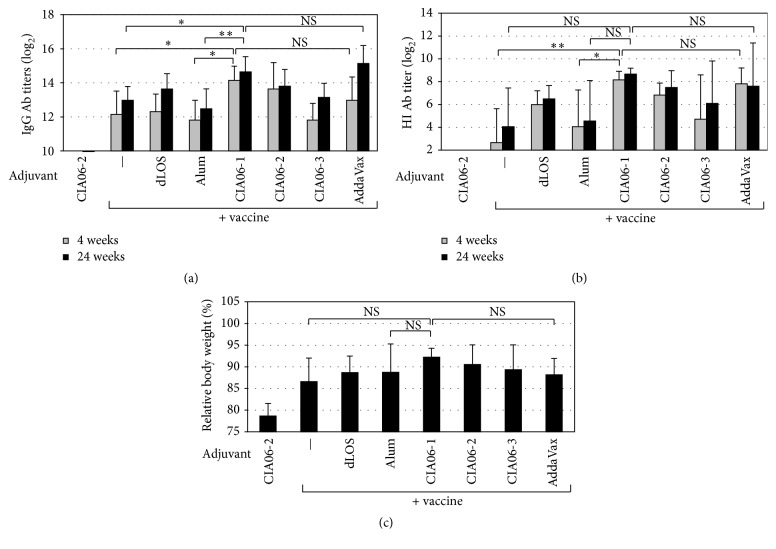
Persistence of protective immunity induced by CIA06-adjuvanted Greenflu-S. Mice (*n* = 6) were immunized twice with Greenflu-S (0.2 *μ*g) alone or in combination with adjuvants. Various ratios of dLOS and alum were used for comparison. CIA06-1 contains 0.25 *μ*g dLOS and 12.5 *μ*g alum, CIA06-2 contains 0.5 *μ*g dLOS and 12.5 *μ*g alum, and CIA06-3 contains 0.5 *μ*g dLOS and 6.25 *μ*g alum. Mice receiving CIA06-2 or Greenflu-S adjuvanted with AddaVax (50 *μ*L) were included as control groups. Sera were obtained 4 and 24 weeks after immunization, and influenza virus-specific IgG antibody titers (a) and HI antibody titers (b) were determined. (c) Mice were challenged with influenza virus 26 weeks after immunization and monitored for mortality and morbidity for 15 days. Body weight changes of mice relative to their initial body weight were calculated for each group at day 5 after infection, when body weight loss was greatest. Statistical differences were analyzed by one-way ANOVA followed by Tukey's multiple comparison test. Results are presented as the GMT ± SD of titers ((a) and (b)) or the means ± SD of values (c) that were obtained from six mice in each group. ^*∗*^ *P* < 0.05 and ^*∗∗*^ *P* < 0.01. NS, not significant.

To determine the persistence of the protective immunity induced by the adjuvanted vaccines, antibody titers were compared 4 and 24 weeks after the second immunization. Mice were immunized with the influenza vaccine alone or in combination with alum or dLOS or combinations of the two at various ratios. Four weeks after immunization, influenza virus-specific antibody titers were highest in the mice that received the vaccine combined with CIA06-1 (12.5 *μ*g alum and 0.25 *μ*g dLOS) (*P* < 0.05 versus the alum-adjuvanted vaccine) (Figure 6(a)). However, changes in the ratio between the two components of CIA06 resulted in lower antibody titers. The antibody titers remained elevated at 24 weeks in all experimental groups. HI antibody titers in the mouse sera revealed a pattern that was similar to the serum IgG antibody titers (Figure 6(b)). To evaluate the protective efficacy of the immunity induced by the adjuvanted vaccines, the mice were challenged 24 weeks after immunization and observed for morbidity and mortality. All experimental groups survived the viral challenge with an exception of the adjuvant control group, all of which died by day 8 (Figure 6(c)). Nevertheless, differences were observed in the percent of body weight change among the groups. Mice given the nonadjuvanted vaccine experienced the highest percent of body weight loss (13.4%). The animals given the CIA06-1 adjuvanted vaccine exhibited the lowest percent of body weight loss (7.8%), and changes in the ratio of the two components resulted in the lower protective activity of CIA06-2 and CIA06-3. For comparison, the AddaVax-adjuvanted vaccine showed 11.8% loss, being similar to the loss of the mice given the alum- or dLOS-adjuvanted vaccine. These data suggested that the CIA06 adjuvant is capable of providing a long-lasting protective immunity against the influenza virus and that the ratio of dLOS and alum is important for achieving the optimal adjuvant effect.

### 3.5. Safety of CIA06-Adjuvanted Greenflu-S against Excessive Inflammatory Response upon Viral Infection

**Figure 7:**
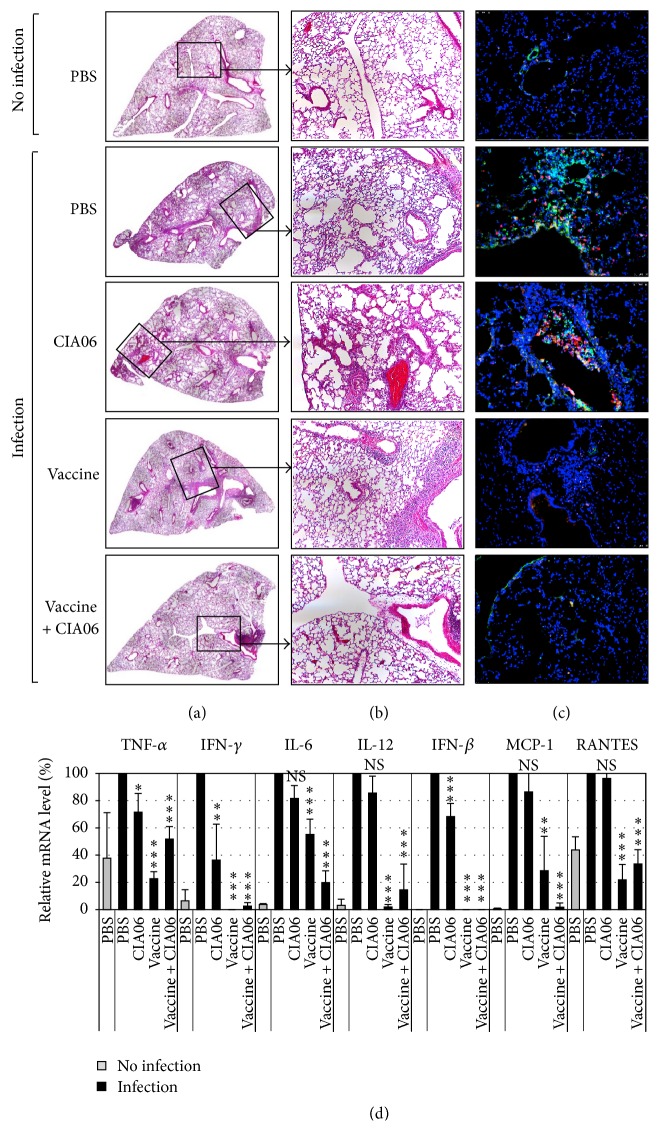
CIA06-adjuvanted Greenflu-S does not induce excessive inflammatory responses in mouse lungs upon influenza infection. Mice (*n* = 3) were immunized twice at a 2-week interval with nonadjuvanted or CIA06-adjuvanted Greenflu-S (0.2 *μ*g). Control mice were given PBS or CIA06. Two weeks later, mice were challenged with influenza virus, and lung samples were collected 4 days after infection. (a) H&E-stained sections of the mouse lung were merged to display a whole lobe of the lung. (b) An original image of the H&E-stained sections of the lung is shown. Magnification 100x. (c) Lung sections were subjected to immunostaining with anti-mouse MPO antibody (green) and anti-influenza A NP antibody (red), followed by staining with DAPI (blue). The images presented are representative of 20 to 45 images from three mice in each group. Magnification 200x. (d) Total cellular RNA was isolated from the lung tissues of each mouse, and mRNA levels of cytokine and chemokine genes were analyzed by semiquantitative RT-PCR and expressed as relative levels to those of the infected PBS control mice. ^*∗*^ *P* < 0.05; ^*∗∗*^ *P* < 0.01; and ^*∗∗∗*^ *P* < 0.001. NS, not significant as compared with the infected PBS control group.

Cytokine storm refers to excessive inflammatory responses induced by viral infection and has been previously associated with a viral respiratory infection in children immunized with the respiratory syncytial virus (RSV) vaccine [18, 19]. In order to determine if the CIA06-adjuvanted influenza vaccine induced excessive cytokine production or harmful inflammation in the lungs of immunized mice following influenza infection, histological examinations of the lungs from the immunized mice were performed in addition to cytokine and chemokine expression assays. In the mice given PBS or CIA06, influenza virus infection resulted in severe lung injuries that were manifested by immune cell infiltration in alveoli, increased alveolar wall thickness, and peribronchial inflammation (Figures 7(a) and 7(b)). Immunostaining of the lung tissues with anti-MPO and anti-influenza A NP antibodies revealed a high degree of neutrophil infiltration and influenza virus surrounding the bronchioles. Neutrophil infiltration and influenza virus production were significantly reduced in mice given the nonadjuvanted vaccine but were only absent in the animals that were immunized with the CIA06-adjuvanted vaccine (Figure 7(c)).

RT-PCR was performed to detect cytokine and chemokine gene expression in the lungs of the infected mice. In the PBS and CIA06 control groups, viral challenge induced a high degree of proinflammatory cytokine mRNA expression, including TNF-*α*, IFN-*γ*, IL-6, IL-12, and IFN-*β*, as well as the expression of chemokines including MCP-1 and RANTES, though cytokine mRNA levels in the CIA06-treated animals were slightly lower (Figure 7(d)). Mice given the adjuvanted or nonadjuvanted vaccine demonstrated significantly lower levels of cytokine and chemokine expression, which were similar to those of uninfected mice with the exception of IL-6 expression. These results indicated that influenza virus infection did not elicit harmfully excessive inflammatory responses in the animals immunized with the CIA06-adjuvanted vaccine.

## 4. Discussion

In this study, we investigated the adjuvant activity of CIA06 on the influenza split vaccine Greenflu-S. The results demonstrated that CIA06 was highly effective in promoting both antibody response and Th1-type cellular response to the influenza vaccine and that the adjuvant effect of CIA06 was more prominent at lower vaccine doses. The CIA06-adjuvanted vaccine at a dose of 0.01 *μ*g vaccine was more effective compared to the 0.2 *μ*g dose of nonadjuvanted vaccine in terms of protective efficacy. This suggests that addition of CIA06 might enable a 20-fold reduction in the antigen dose required for optimal protection. In this study, the CIA06-1 consisting of 0.25 *μ*g dLOS and 12.5 *μ*g alum demonstrated higher efficacy compared to CIA06-2 (0.5 *μ*g dLOS and 12.5 *μ*g alum) or CIA06-3 (0.5 *μ*g dLOS and 6.25 *μ*g alum). Hence, this data suggests that the ratio of the two components is critical to achieving optimal adjuvant efficacy, which was consistent with our previous observations with HPV vaccine [13].

Adults over the age of 65 years are more vulnerable to influenza infection and its complications; however, antibody responses and the protective efficacy of the influenza vaccine are lower in this population [20]. In addition, elderly adults tend to have low T cell responses following vaccination, which may further contribute to reduced vaccine protective efficacy [20, 21]. To achieve optimal immune responses in elderly individuals, a high-dose vaccine containing four times more antigen than the standard dose vaccine was adopted [22]. Also, the use of adjuvants was explored as a means to overcome poor immune responses to influenza vaccines in the elderly, and the results indicated potential benefits [23, 24]. In this study, mice immunized with the CIA06-adjuvanted influenza vaccine exhibited significantly higher CD4^+^ and CD8^+^ T cell IFN-*γ* secretion as well as the elevated serum IgG antibody titers compared with mice that received the nonadjuvanted vaccine, suggesting the potential benefits of the CIA06-adjuvanted influenza vaccine in the elderly. The influenza vaccine adjuvanted with CIA06 at 1/40 of human dose induced the serum IgG and HI antibody titers comparable to that adjuvanted with AddaVax at 1/5 of human dose and was more protective against influenza virus infection. While the oil-in-water emulsion adjuvant promotes CD4^+^ T cell responses [25], CIA06 is capable of enhancing both CD4^+^ and CD8^+^ T cell responses. Further, cellular immunity is known to provide broad cross-protection against non-vaccine type influenza strains [26, 27] and reduce illness severity in humans [28]; thus, effective induction of cellular immunity by CIA06 could contribute to the protection of the adjuvanted vaccine against antigenically drifted strains.

Alum is the most widely used adjuvant for human vaccines. In this study, we compared the adjuvant activity of CIA06 and alum on the influenza vaccine. Mice given the CIA06-adjuvanted vaccine showed significantly higher serum IgG and HI antibody titers compared to those given the alum-adjuvanted vaccine only when 0.05 *μ*g vaccine dose, but not 0.2 *μ*g dose, was used for immunization. However, the antibody titers of the mice immunized with the alum-adjuvanted vaccine at 0.2 *μ*g dose significantly decreased by 24 weeks after immunization while those immunized with the CIA06-adjuvanted vaccine maintained the initial titers, demonstrating that CIA06 is more effective than alum in providing a long term immunity of the vaccine.

In the 1960s, RSV vaccine containing formalin-inactivated RSV caused severe inflammatory responses in vaccinated infants following natural RSV infection [18]. Subsequent research determined that the RSV fusion-protein (F-protein) acts as a TLR4 agonist and causes excessive Th2-type immune responses upon RSV infection in FI-RSV vaccinated mice [19, 29, 30]. Similarly, infection with highly pathogenic influenza virus induces an acute lung injury, leading to acute respiratory distress syndrome (ARDS), which is mediated through TLR4 signaling as a key disease pathway [31]. Excessive macrophage and neutrophil infiltration in the lungs following influenza virus infection cause ARDS [31]. Reports indicated that the addition of MPL to FI-RSV mitigated the adverse immune response to RSV infection in the lung and was associated with a reduction in Th2-type cytokine expression [32, 33]. In this study, CIA06 induced a Th1-type-predominant immune response to the influenza vaccine and reduced the production of Th2-type cytokines. In fact, lung histology and cytokine and chemokine gene expression demonstrated that the CIA06-adjuvanted vaccine reduced hypercytokinemia and hyperneutrophilia in the lungs following natural infection.

In summary, the results of the current study demonstrated that CIA06 effectively enhanced the protective efficacy of the H1N1 pandemic influenza virus vaccine, especially when low vaccine doses were used for immunization, and that the CIA06-adjuvanted vaccine does not induce harmful inflammatory responses following viral infection in vaccinated animals. These data suggest that CIA06 might be a feasible solution to potentiating the efficacy of influenza vaccine for elderly population as well as conserving vaccine supplies in the event of a vaccine shortage during an influenza pandemic.

## Supplementary Material

The nucleotide sequences of the primers of each gene used for RT-PCR in this study are shown in the table.

## References

[1] O'Hagan D. T., Fox C. B. (2015). Are we entering a new age for human vaccine adjuvants?. *Expert Review of Vaccines*.

[2] Brown L. E. (2010). The role of adjuvants in vaccines for seasonal and pandemic influenza. *Vaccine*.

[3] Clark T. W., Pareek M., Hoschler K. (2009). Trial of 2009 influenza A (H1N1) monovalent MF59-adjuvanted vaccine. *The New England Journal of Medicine*.

[4] Black S. (2015). Safety and effectiveness of MF-59 adjuvanted influenza vaccines in children and adults. *Vaccine*.

[5] Garçon N., Vaughn D. W., Didierlaurent A. M. (2012). Development and evaluation of AS03, an Adjuvant System containing *α*-tocopherol and squalene in an oil-in-water emulsion. *Expert Review of Vaccines*.

[6] Launay O., Duval X., Fitoussi S. (2013). Extended antigen sparing potential of AS03-adjuvanted pandemic H1N1 vaccines in children, and immunological equivalence of two formulations of AS03-adjuvanted H1N1 vaccines: results from two randomised trials. *BMC Infectious Diseases*.

[7] Baldwin S. L., Shaverdian N., Goto Y. (2009). Enhanced humoral and type 1 cellular immune responses with Fluzone® adjuvanted with a synthetic TLR4 agonist formulated in an emulsion. *Vaccine*.

[8] Cho Y. J., Ahn B. Y., Lee N. G., Lee D. H., Kim D.-S. (2006). A combination of *E. coli* DNA fragments and modified lipopolysaccharides as a cancer immunotherapy. *Vaccine*.

[9] Han J. E., Wui S. R., Kim K. S., Cho Y. J., Cho W. J., Lee N. G. (2014). Characterization of the structure and immunostimulatory activity of a vaccine adjuvant, de-O-acylated lipooligosaccharide. *PLoS ONE*.

[10] Song E. S., Park S. A., Kim S. H. (2007). Adjuvant effect of CIA07, a combination of Escherichia coli DNA fragments and modified lipopolysaccharides, on the immune response to hepatitis B virus surface antigen. *FEMS Immunology and Medical Microbiology*.

[11] Park S. A., Song E. S., Cho Y. J. (2007). Immune responses of mice to influenza subunit vaccine in combination with CIA07 as an adjuvant. *Microbiology and Immunology*.

[12] Han J. E., Kim H. K., Park S. A. (2010). A nontoxic derivative of lipopolysaccharide increases immune responses to Gardasil® HPV vaccine in mice. *International Immunopharmacology*.

[13] Han J. E., Wui S. R., Park S. A. (2012). Comparison of the immune responses to the CIA06-adjuvanted human papillomavirus L1 VLP vaccine with those against the licensed HPV vaccine Cervarix ™ in mice. *Vaccine*.

[14] Wui S. R., Kim H. K., Han J. E. (2011). A combination of the TLR4 agonist CIA05 and alum promotes the immune responses to *Bacillus anthracis* protective antigen in mice. *International Immunopharmacology*.

[15] Wui S. R., Han J. E., Kim Y. H., Rhie G.-E., Lee N. G. (2013). Increased long-term immunity to Bacillus anthracis protective antigen in mice immunized with a CIA06B-adjuvanted anthrax vaccine. *Archives of Pharmacal Research*.

[16] Lee C.-H., Tsai C.-M. (1999). Quantification of bacterial lipopolysaccharides by the purpald assay: measuring formaldehyde generated from 2-keto-3-deoxyoctonate and heptose at the inner core by periodate oxidation. *Analytical Biochemistry*.

[17] Shim B.-S., Choi J.-A., Song H.-H. (2013). Sublingual administration of bacteria-expressed influenza virus hemagglutinin 1 (HA1) induces protection against infection with 2009 pandemic H1N1 influenza virus. *Journal of Microbiology*.

[18] Kim H. W., Canchola J. G., Brandt C. D. (1969). Respiratory syncytial virus disease in infants despite prior administration of antigenic inactivated vaccine. *American Journal of Epidemiology*.

[19] Waris M. E., Tsou C., Erdman D. D., Zaki S. R., Anderson L. J. (1996). Respiratory synctial virus infection in BALB/c mice previously immunized with formalin-inactivated virus induces enhanced pulmonary inflammatory response with a predominant Th2-like cytokine pattern. *Journal of Virology*.

[20] McElhaney J. E. (2011). Influenza vaccine responses in older adults. *Ageing Research Reviews*.

[21] McElhaney J. E., Xie D., Hager W. D. (2006). T cell responses are better correlates of vaccine protection in the elderly. *Journal of Immunology*.

[22] DiazGranados C. A., Dunning A. J., Jordanov E., Landolfi V., Denis M., Talbot H. K. (2013). High-dose trivalent influenza vaccine compared to standard dose vaccine in elderly adults: safety, immunogenicity and relative efficacy during the 2009-2010 season. *Vaccine*.

[23] Frey S. E., Reyes M. R. A.-D. L., Reynales H. (2014). Comparison of the safety and immunogenicity of an MF59®-adjuvanted with a non-adjuvanted seasonal influenza vaccine in elderly subjects. *Vaccine*.

[24] Yang W. H., Dionne M., Kyle M. (2013). Long-term immunogenicity of an AS03-adjuvanted influenza A(H1N1)pdm09 vaccine in young and elderly adults: an observer-blind, randomized trial. *Vaccine*.

[25] Galli G., Medini D., Borgogni E. (2009). Adjuvanted H5N1 vaccine induces early CD4+ T cell response that predicts long-term persistence of protective antibody levels. *Proceedings of the National Academy of Sciences of the United States of America*.

[26] Altenburg A. F., Rimmelzwaan G. F., de Vries R. D. (2015). Virus-specific T cells as correlate of (cross-)protective immunity against influenza. *Vaccine*.

[27] Soema P. C., van Riet E., Kersten G., Amorij J. P. (2015). Development of cross-protective influenza vaccines based on cellular responses. *Frontiers in Immunology*.

[28] Sridhar S., Begom S., Bermingham A. (2013). Cellular immune correlates of protection against symptomatic pandemic influenza. *Nature Medicine*.

[29] Kurt-Jones E. A., Popova L., Kwinn L. (2000). Pattern recognition receptors TLR4 and CD14 mediate response to respiratory syncytial virus. *Nature Immunology*.

[30] Imai Y., Kuba K., Neely G. G. (2008). Identification of oxidative stress and toll-like receptor 4 signaling as a key pathway of acute lung injury. *Cell*.

[31] Narasaraju T., Yang E., Samy R. P. (2011). Excessive neutrophils and neutrophil extracellular traps contribute to acute lung injury of influenza pneumonitis. *The American Journal of Pathology*.

[32] Boukhvalova M. S., Prince G. A., Soroush L., Harrigan D. C., Vogel S. N., Blanco J. C. G. (2006). The TLR4 agonist, monophosphoryl lipid A, attenuates the cytokine storm associated with respiratory syncytial virus vaccine-enhanced disease. *Vaccine*.

[33] Prince G. A., Denamur F., Deschamps M. (2001). Monophosphoryl lipid A adjuvant reverses a principal histologic parameter of formalin-inactivated respiratory syncytial virus vaccine-induced disease. *Vaccine*.

