# Supplementary material for: A De-O-acylated Lipooligosaccharide-Based Adjuvant System Promotes Antibody and Th1-Type Immune Responses to H1N1 Pandemic Influenza Vaccine in Mice
Source: Biomed Res Int. 2016 Nov 7;2016:3713656. doi: 10.1155/2016/3713656 (PMC5116492; doi:10.1155/2016/3713656)
Supplement: Supplementary file 1 — The nucleotide sequences of the primers of each gene used for RT-PCR in this study are shown in the table. [file 3713656.f1.pdf]

Supporting Information

Table S1. Primers used for forward and reverse RT-PCR.

Target gene	Accession number	PCR primer sequences (5′–3′)	Product size (bp)
β-actin	NM_007393	F 5′- TGGAATCCTGTGGCATCCATGA -3′<br>R 5′- TCTTGATCTTCATGGTGCTAGG -3′	180
TNF-α	NM_013693	F 5′- ATGAGCACAGAAAGCATGATC -3′<br>R 5′- GTTCAGTAGACAGAAGAGCG -3′	156
IFN-γ	NM_008337	F 5′- GGATATCTGGAGGAACTGGCAA -3′<br>R 5′- CCTCAAACTTGGCAATACTCATG -3′	209
IL-6	NM_031168	F 5′- CTTCCATCCAGTTGCCTTCTT -3′<br>R 5′- ACGATTTCCCAGAGAACATGT -3′	165
IL-12	NM_008352	F 5′- CAAGACTTTCCTGAAGTGTGAAG -3′<br>R 5′- AATGGCGTCTCTGTCTGCAGAGAA -3′	162
IFN-β	NM_010510	F 5′- ACTATAAGCAGCTCCAGCTC -3′<br>R 5′- AAGACATTCTGGAGCATCTC -3′	184
MCP-1	NM_011333	F 5′- ACTCACCTGCTGCTACTCAT -3′<br>R 5′- TCAGCACAGACCTCTCTCTT -3′	138
RANTES	NM_013653	F 5′- ACCATATGGCTCGGACACCA -3′<br>R 5′- TTCTCTGGGTTGGCACACAC -3′	162
